# Zinc Supplementation Promotes a Th1 Response and Improves Clinical Symptoms in Fewer Hours in Children With Pneumonia Younger Than 5 Years Old. A Randomized Controlled Clinical Trial

**DOI:** 10.3389/fped.2019.00431

**Published:** 2019-11-14

**Authors:** Jorge Alberto Acevedo-Murillo, Miguel Leonardo García León, Verónica Firo-Reyes, Jorge Luis Santiago-Cordova, Alejandra Pamela Gonzalez-Rodriguez, Rosa María Wong-Chew

**Affiliations:** ^1^Hospital Pediátrico de Coyoacán, Mexico City, Mexico; ^2^Laboratorio de Investigación en Enfermedades Infecciosas, División de Investigación, Facultad de Medicina, Universidad Nacional Autónoma de México, Mexico City, Mexico; ^3^Servicio de Pediatría, Hospital General de México Dr. Eduardo Liceaga, Mexico City, Mexico

**Keywords:** zinc supplementation, pneumonia, children, immune response, Th1 cytokines

## Abstract

**Background:** Pneumonia caused 704,000 deaths in children younger than 5 years in 2015. Zinc is an important micronutrient due to its role in immune function. Since 2004, WHO recommends zinc supplementation for children with diarrhea to shorten the duration and decrease severity. Zinc supplementation for children with pneumonia is controversial.

**Methods:** A randomized controlled clinical trial was conducted, and 103 children 1 month to 5 years old with pneumonia were included. Zinc or placebo was given during hospitalization. Clinical symptoms were recorded, and a blood draw was obtained to determine serum zinc levels, lymphoproliferation, and cytokines at hospitalization and at discharge of the patient; a nasal wash was obtained to detect viral or bacterial pathogens by multiplex RT-PCR.

**Results:** Zinc supplementation improved in fewer hours the clinical status (76 ± 7 vs. 105 ± 8, *p* = 0.01), the respiratory rate (37 ± 6 vs. 57 ± 7, *p* = 0.04), and the oxygen saturation (53 ± 7 vs. 87 ± 9, *p* = 0.007) compared to the placebo group. An increase in IFNγ and IL-2 after treatment in the zinc group was observed.

**Conclusions:** Zinc supplementation improved some clinical symptoms in children with pneumonia in fewer hours and induced a cellular immune response.

**Clinical Trial Registration:** The trial was retrospectively registered in ClinicalTrials.gov, identifier NCT03690583, URL https://clinicaltrials.gov/ct2/show/NCT03690583?term=zinc+children&cond=Pneumonia&draw=2&rank=1.

## Background

Great progress has been achieved for Millennium Development Goal 4: “to reduce child mortality rate by two-thirds.” The number of deaths in children under 5 decreased almost 40%, from 12.4 million in 1990 to 7.6 million in 2010 and by 51% from 2000 2015 (1, 2). Although infectious diseases are still a major disease burden—pneumonia, diarrhea, and malaria accounted for 30% of deaths in children younger than 5 years old in 2010—it is estimated that lower respiratory tract infections caused 2.74 million deaths in 2015, of which 704,000 were in children younger than 5 years old (3), and 652,572 deaths in 2016 (4). A decrease of 22% was observed in episodes of childhood pneumonia in young children from 178 million in 2000 to 138 million in 2015 and in the number of deaths from pneumonia from 1.7 million in 2000 to 0.9 million in 2015 (2, 5).

Malnutrition is a predisposing factor for recurrent and severe infections and includes zinc deficiency. Approximately 2 billion people in developing countries suffer from zinc deficiency, mainly due to malnutrition. The estimated prevalence of inadequate zinc intake varies from 7.5% in high-income regions to 30% in South Asia, and in the case of Mexico, the estimated prevalence of inadequate zinc intake is 15–25% (6). Zinc is a micronutrient that plays important roles in cell proliferation, differentiation, and immune function, acting as a cofactor for proteins and structural and catalytic functions of enzymes and transcription factors (7). Zinc promotes neutrophil extracellular traps, induces cell-mediated immunity over humoral immunity by regulating factors or differentiation, modulates the proinflammatory response by targeting NFκB, controls oxidative stress, and regulates inflammatory cytokines (8). Zinc deficiency promotes thymic atrophy and lymphopenia and decreases innate and adaptive immunity: it impairs host defense by neutrophils and natural killer cells, phagocytosis, intracellular killing activity, and cytokine production by macrophages and alters proliferation, cytokine production, and antibody secretion of T and B cells (9).

The World Health Organization (WHO) and Unicef recommend, since 2004, zinc supplementation along with oral rehydration to treat acute diarrhea because of the benefit of zinc in shortening and decreasing the severity reported in numerous randomized placebo-controlled trials (10).

In the case of zinc supplementation for children with pneumonia, the results are controversial. Some studies report a marginal improvement in hospital stay (11). Others report no differences in tachypnea, respiratory distress, or days for resolution of pneumonia compared to controls (12–16). And others report improvement in the clinical outcome and duration of hospital stay (17, 18) and prevention of mortality, incidence, and prevalence (19–23), including children with HIV in whom a decrease in days of hospital stay was observed (24). Some report higher basal zinc and better nutritional status as an effect of zinc on time to clinical resolution (25). A study in Uganda reported a decrease in mortality from 12 to 4% in children 6 months to 5 years old with pneumonia, with a decrease in the relative risk of 0.67 in mortality (26).

Cell-mediated immunity plays an important role in the host defense against viruses and bacteria; zinc supplementation in children with some degree of malnutrition and with a severe infection could improve the immune response to an acute infection. There are few reports of the assessment of the immune response in children with pneumonia who receive zinc supplementation along with the standard pneumonia treatment and the detection of the etiological agents by multiplex PCR.

The aim of this study was to evaluate the immunomodulatory effect of zinc supplementation and the correlation of the clinical response and etiological agent in children with pneumonia younger than 5 years old.

## Methods

### Study Population

Previously healthy children between 1 month and 5 years old with a clinical and/or radiological diagnosis of pneumonia admitted to the Hospital General de Mexico Dr. Eduardo Liceaga and the Hospital Pediátrico de Coyoacán were included. The very-low-income population of Mexico City and the metropolitan area attend these hospitals. The case definition for pneumonia was a child with signs and symptoms including fever, cough, tachycardia, tachypnea, dyspnea, respiratory distress, rales, and/or a radiographic pattern of pneumonia (27–29). Inclusion criteria were previously healthy children with the case definition of pneumonia. Exclusion criteria were zinc intake in the 2 previous weeks, history of cardiac and/or pulmonary disease including asthma, and prematurity.

The study was approved by the Institutional Review Boards of the Faculty of Medicine, Universidad Nacional Autónoma de México (036-2012), Hospital General de México (DI/13/505/05/026), and Hospital Pediátrico de Coyoacán (3020010114); written consent was obtained from parents or guardians. The procedures followed were in accordance with the ethical standards of the Helsinki Declaration.

### Randomization and Masking

A triple-blinded randomized controlled clinical trial was conducted. The principal investigator generated the random allocation sequence. The pediatricians who recruited the patients, the patients and their mothers, and the laboratory technicians who performed the immunological and molecular tests did not know who received zinc or placebo.

At the time of admission to the hospital, parents or guardians were invited to have their children with pneumonia participate in the study and signed an informed consent. Children were randomly assigned 1:1 using a list of random numbers generated by Epistats to receive zinc sulfate (10 mg for children younger than 1 year old, 20 mg for children older than 1 year old) or placebo (20 mg of glucose). The list was generated by the principal investigator, and the participants were assigned to each group by the pediatricians recruiting the patients.

### Study Procedures

Zinc and placebo contained in identical paper containers marked as A or B were diluted in 1 ml of distilled water and administered every day orally throughout the duration of hospitalization. Both substances looked like white powder inside the envelopes, and when they were diluted in distilled water, they also looked the same. At the inclusion of the child demographic, clinical characteristics, laboratory workup, a chest x-ray, and risk factors for pneumonia were obtained, and during the follow-up, clinical records including heart rate, oxygen saturation, respiratory distress, temperature, cough, rales, wheezing, and feeding capacity were recorded periodically six times a day until the discharge of the patient in a special format designed for this study. The nurses record the vital signs two times per shift in a hospital sheet, and there are three shifts per day. The clinical records were taken from those records and verified by the pediatricians. All the patients received the standard treatment protocol for children with pneumonia, which includes oxygen supplementation and intravenous fluid, and according to the criteria of the consultant physician, penicillin or ampicillin was administered or oseltamivir if influenza was suspected. Two blood draws were obtained, one when the child was admitted to the hospital and another one the day of discharge to perform lymphoproliferation and cytokine assays and to obtain sera, which was frozen at −70°C until processing to determine serum zinc levels.

Nasal washes were also obtained the day of hospitalization by instilling 1 ml of physiological saline solution through a sterile feeding tube connected to a syringe and aspirating the solution from each nostril, placing the content into viral medium, and storing at −70°C until processing to detect viral or bacterial genetic material. The nasal washes and blood samples were processed at the Unidad de Investigación en Medicina Experimental, Facultad de Medicina, UNAM.

### Zinc Detection

Zinc in the sera of the patients at hospitalization and discharge was determined using a commercial kit (Zinc Monoliquid Fortress-Diagnostics, USA). Fifty microliters of serum was added to 1,000 μl of the reagent (5-Br-PAPS [2-(5-bromo-2-pyridylazo)-5-(N-propyl-N-sulfopropylamino)-phenol]) incubated for 5 min at 37°C and compared to the standard 200 μg/dl (30.6 mol/L). The sample was read at a wavelength of 560 nm in a Genesys 10S UV-Vis Spectrophotometer (Thermo Scientific); the signal intensity is directly proportional to the amount of zinc. The normal serum zinc is 63.8–110 μg/dl (9.8–16.8 μmol/L).

### RT-PCR Technique

The nasal washes were processed using the Anyplex II RV16 kit (Seegene, Seoul, South Korea). The nucleic acid extraction was performed manually using the Ribo_spin GeneAll vRD kit (GeneAll Biotechnology, Seoul, South Korea); before extraction, an internal control of the Anyplex kit was added to each sample. The cDNA was synthesized from extracted RNA with the cDNA Synthesis Kit Premix (Seegene). Subsequently, real-time PCR was carried out in the CFX96 equipment (Bio-Rad) through the Anyplex II RV16 Detection kit, which uses TOCE technology. Amplified respiratory viruses were visualized using the Seegene Viewer software. The Anyplex RV16 kit has the capacity to detect 14 RNA viruses and 2 DNA viruses: respiratory syncytial virus types A and B; influenza virus types A and B; parainfluenza virus types 1–4; adenovirus; metapneumovirus; coronavirus OC43, 229E, and NL63; rhinovirus types A, B, and C; enterovirus; and bocavirus. Bacterial genetic material was detected in nasal washings using the Seeplex RV15 ACE detection kit (Seegene, Seoul Korea) with simultaneous detection with a multiplex PCR using the DPO technology. The kit detects *Streptococcus pneumoniae, Haemophilus influenzae, Chlamydophila pneumoniae, Legionella pneumophila, Bordetella pertussis*, and *Mycoplasma pneumoniae*.

### Lymphoproliferation Assay

Fresh peripheral blood mononuclear cells (PBMCs) were obtained from whole blood by Ficoll-Hypaque density gradient. PBMCs were placed in a 96-well-plate at 3.0 × 10^5^ cells per well in RMPI 1640 medium (Gibco, Gaithersburg, MD) with 10% normal human sera (Sigma, St. Louis, MO); 5 μg of concanavalin A (Sigma-Aldrich) was added, and cells were incubated at 37°C and 5% CO_2_ for 5 days. EdU Click-iT Alexa Fluor 488 (Invitrogen) was added for 6 h, and cells were acquired by flow cytometry using the FACSCanto II cytometer (Becton Dickinson); the lymphoproliferation was analyzed using the FacsDiva software (Becton Dickinson) determined by the difference in fluorescence intensity means (MiFID) of the control and ConA wells.

### Cytokine Assays

PBMCs were placed in a 96-wells plate at 3.0 x 10^5^ cells per well in RMPI 1640 medium (Gibco, Gaithersburg, MD) with 5% normal human sera (Sigma, St. Louis, MO); 3 μl of Cytostim (Myltenyi Biotec) or medium as negative control was added and incubated at 37°C and 5% CO_2_ for 6 h, and supernatants of the wells were collected. Cytokines were determined using the Cytometric Bead Array Human Th1/Th2 kit (Becton Dickinson) following the manufacturer's instructions, which includes IL-2, IL-4, IL-6, IL-10, TNF-alpha, and IFN-gamma obtained by flow cytometry (FACSCanto II) and analyzed with the FCAP Array software (Becton Dickinson).

### Sample Size Calculation and Statistical Analysis

Sample size calculation was based on the dependent variable time in hours for improvement using G power to calculate the difference between two independent means. The variance and the difference were obtained from a previous publication (30), with an effect size of 0.5, a power of 80%, and alpha of 0.05, obtaining a sample size of 51 per group, 102 total. Frequencies and proportions of clinical and demographic variables were determined by descriptive statistics. Student *t* and paired *t*-test were used to contrast quantitative variables; chi square was used to contrast qualitative variables. Malnutrition was calculated by Z score and categorized as presence or absence for analysis. Because the placebo group were younger and smaller, in order to detect a bias, a covariance analysis (ANCOVA) was done adjusting for age, weight, height, body mass index (BMI), vaccination status, and nutrition status the dependent variables (clinical improvement, normalization of respiratory rate, normalization of oxygen saturation). A *p* < 0.05 was considered statistically significant.

## Results

**Figure 1:**
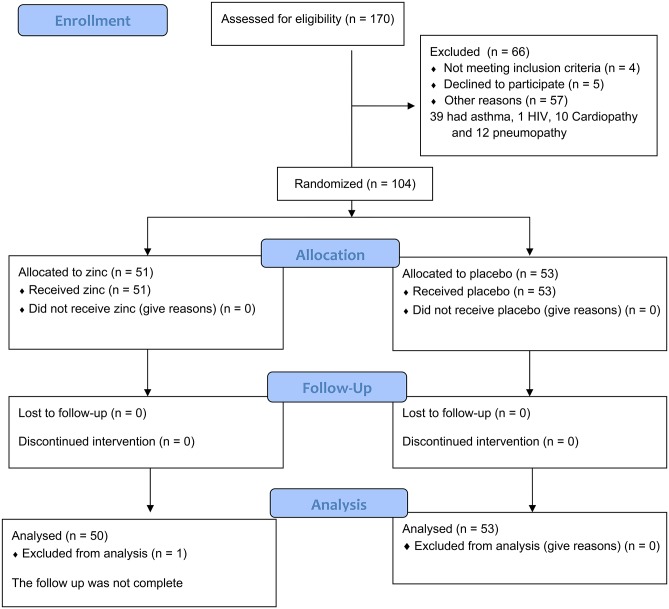
CONSORT flow diagram of the inclusion and analysis of the patients.

One hundred and seventy patients with pneumonia younger than 5 years old were detected at the participating institutions from January 2014 to February 2016; 62 were not eligible because 39 had asthma, 1 HIV infection, 10 cardiopathy, and 12 pneumopathy, and 5 parents declined to participate. Of those eligible and whose parents accepted to have their children participate, 51 received zinc and 53 placebo. All the participants were included in the analysis because there were no losses, except for one patient in the zinc group who did not have the follow-up information and the blood draw. The patients were followed at the hospitals during their treatment (Figure 1 and Supplementary Table 1).

### Demographic and Clinical Characteristics

The mean ± SE age in the zinc group was 23 ± 2.2 months compared to 18 ± 2.2 in the placebo group (*p* = 0.09). The children in the placebo group had a lower weight and height compared to the zinc group (9.4 ± 0.5 vs. 11.1 ± 0.5, *p* = 0.01, and 74.9 ± 2.2 vs. 81.4 ± 2.3, *p* = 0.05, respectively), and there were more males in the placebo group compared to the zinc group (64 vs. 46%, *p* = 0.04, respectively). The baseline clinical symptoms were comparable (according to the WHO pneumonia classification)—pneumonia (66 vs. 62%), severe pneumonia (32 vs. 34%, *p* = 0.83), oxygen saturation (85 ± 0.7 vs. 84 ± 0.7, *p* = 0.36), and respiratory rate (mean ± SE, 43 ± 1.4 vs. 45 ± 1.5, *p* = 0.54)—between the zinc and the placebo group, respectively. The predominant radiographic pattern was interstitial (82 vs. 71%), followed by alveolar (9 vs. 13%) and mixed (2 vs. 11%) (*p* = 0.26), in the zinc and the placebo group. Also comparable was the percentage of children with rales, fever, cough, respiratory distress, rhinorrhea, vomit, nasal flare, and costal retraction by group. No differences in the percentage of symptomatic, antibiotic, or antiviral treatment between groups were observed. Penicillin is used as empiric treatment for CAP in children at the Hospital Pediatrico de Coyoacan and ampicillin at the Hospital General de México, according to local hospital guidelines based on sensitivity patterns (Table 1).

**Table 1:** Baseline demographic and clinical characteristics of children with pneumonia who received zinc supplementation and placebo.

	**Zinc *n* = 50**	**Placebo *n* = 53**	***p***
Age (months), mean ± SE	23 ± 2.2	18 ± 2.2	0.09
Weight (kg), mean ± SE	11.15 ± 0.5	9.4 ± 0.5	0.01
Height (cm), mean ± SE	81.4 ± 2.3	74.9 ± 2.2	0.05
Body mass index (BMI), mean ± SE	16.5 ± 0.4	15.9 ± 0.3	0.35
Female, *n* (%)	27 (54)	19 (36)	0.04
Male, *n* (%)	23 (46)	34 (64)	
Pneumonia, *n* (%)	33 (66)	33 (62)	0.83
Severe pneumonia, *n* (%)	16 (32)	18 (34)	
Very severe disease, *n* (%)	1 (2)	2 (4)	
O_2_ saturation, mean ± SE	85 ± 0.7	84 ± 0.7	0.36
Respiratory rate, mean ± SE	43 ± 1.4	45 ± 1.5	0.54
Respiratory distress, %	59	63	0.46
Rales, %	96	94	0.52
Fever, %	37	40	0.46
Cough, %	65	76	0.2
Rhinorrhea, %	49	53	0.45
Vomit, %	11	10	0.62
Nasal flare, %	5	3	0.49
Costal retraction, %	8	0	0.11
X-ray normal pattern, %	7	5	0.26
Alveolar pattern, %	9	13	
Interstitial pattern, %	82	71	
Mixed pattern, %	2	11	
Symptomatic treatment^+^, *n* (%)	6 (12)	6 (11)	0.86
Antibiotics^++^, *n* (%)	43 (86)	45 (85)	
Antibiotics and antivirals, *n* (%)	1 (2)	2 (4)	
Zinc pre-treatment, mcg/dl (mean ± SE)	23 ± 1.8^*^	21 ± 1.9^**^	0.34
Zinc post-treatment, mcg/dl (mean ± SE)	33 ± 4^*^	29 ± 2.8^**^	0.45

+ Symptomatic treatment includes oxygen supplementation, intravenous fluids, and antipyretic and anti-inflammatory agents;

++ penicillin or ampicillin;

* zinc group pre- vs. post-zinc levels p = 0.003;

** *placebo group pre- vs. post-zinc levels p = 0.08*.

Zinc levels were below the normal range (63–110 mcg/mL) at baseline (23 ± 1.8 vs. 21 ± 1.9, *p* = 0.34) and after treatment (33 ± 4 vs. 29 ± 2.8, *p* = 0.45), and no differences were found between zinc and placebo, respectively. Both groups increased zinc levels after supplementation, but the placebo group was not significant. Although the zinc group showed a statistically significant increase after supplementation (*p* = 0.003), it did not reach the normal serum level reported (Table 1). No side effects were reported with zinc or placebo supplementation.

### Risk Factors

Both groups were comparable for the following risk factors—low income (96 vs. 94%, *p* = 0.52), malnutrition (23 vs. 28%, *p* = 0.41), domestic smoking (32 vs. 36%, *p* = 0.39), absence of breastfeeding (24 vs. 21%, *p* = 0.88), and overcrowding (62 vs. 58%, *p* = 0.4), in the zinc and the placebo group, respectively. The proportion of children with an incomplete vaccination schedule was higher in the placebo group (50 vs. 30%, *p* = 0.04), and there were more children attending daycare centers in the zinc group (32 vs. 11%, *p* = 0.01) (Table 2).

**Table 2:** Risk factors for pneumonia and viral or bacterial detection.

**Risk factors**	**Zinc *n* = 50**	**Placebo *n* = 53**	***p***
Low income, *n* (%)	48 (96)	50 (94)	0.52
Middle income, *n* (%)	2 (4)	3 (6)	
Malnutrition *n* (%)	12 (23)	15 (28)	0.41
Incomplete vaccination schedule, *n* (%)	15 (30)	25 (50)	0.04
Domestic smoking, *n* (%)	16 (32)	19 (36)	0.39
Daycare assistance, *n* (%)	16 (32)	6 (11)	0.01
Formula, *n* (%)	12 (24)	11 (21)	0.88
Breastfeed, *n* (%)	22 (45)	23 (44)	
Mixed, *n* (%)	15 (31)	18 (35)	
Overcrowding, *n* (%)	31 (62)	30 (58)	0.4
Biomass use, *n* (%)	3 (6)	2 (4)	0.5
**Viral and bacterial detection**			
Negative, *n* (%)	8 (16)	3 (6)	0.57
Viral single agent, *n* (%)	10 (20)	12(23)	
Viral coinfection, *n* (%)	7 (14)	7 (13)	
Viral and bacterial coinfection, *n* (%)	22 (44)	27 (52)	
Bacterial single agent, *n* (%)	3 (6)	3 (6)	

*Children who received placebo had a higher percentage of incomplete vaccination schedule, and children in the zinc group had higher daycare attendance*.

### Viral and Bacterial Detection

The percentage of viral and bacterial detection was comparable among groups−20 vs. 23% one virus, 14 vs. 13% viral coinfection, 44 vs. 52% viral and bacterial coinfection, 6 vs. 6% bacteria (*p* = 0.57), in the zinc and placebo groups, respectively (Table 2).

**Figure 2:**
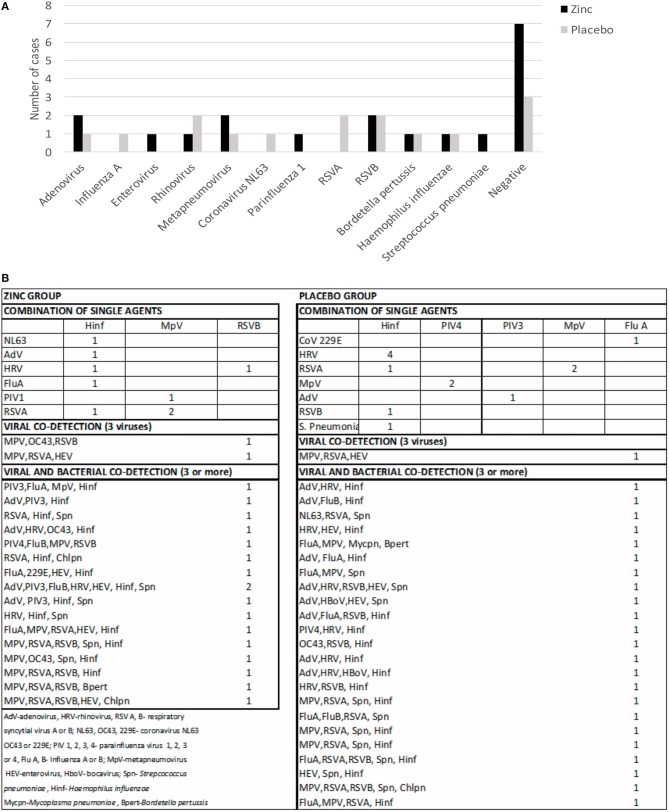
Viral and bacterial detection in children with pneumonia who received zinc or placebo. **(A)** Detection of single agents by group. **(B)** Number of combined detection of virus–virus, bacteria–bacteria, or virus–bacteria by group.

Distribution of virus and bacteria in both groups is shown in Figure 2. RSV A and B, rhinovirus, and metapneumovirus were the most frequent detected viruses in both groups as single agents or in combination. *H. influenzae* and *S. pneumoniae* were the most frequently detected bacteria in both groups as single agents or in combination. The incidence of pathogens detected was similar in both groups. There were more than three pathogens in the majority of patients in the zinc (58%) and the placebo (65%) group, respectively (Figure 2B). Analysis of only virus (Supplementary Table 2) or only bacteria showed no differences in frequencies among groups.

### Comparison of the Time for Improvement

The clinical improvement (mean in hours of the combination of all the clinical variables) (76 ± 7 vs. 105 ± 8, *p* = 0.01), the normalization of the respiratory rate (37 ± 6 vs. 57 ± 7, *p* = 0.04), and the normalization of oxygen saturation (53 ± 7 vs. 87 ± 9, *p* = 0.007) measured in hours was faster in the zinc group compared to the placebo group and statistically significant. In the analysis of covariance adjusting the effect of zinc for age (because the placebo group were younger), a *p* of 0.01 was found for time for clinical improvement, 0.07 for respiratory rate, and 0.009 for oxygen saturation, meaning that the younger age in the placebo group was not a bias and had no effect for the better response in the zinc group. Also, weight, height, BMI, gender, vaccination status, and nutrition status had no effect in the covariance analysis on the dependent variables.

There were no statistically significant differences in the normalization of respiratory distress (46 ± 4 vs. 56 ± 6, *p* = 0.21), normalization of temperature (6 ± 1 vs. 7 ± 2, *p* = 0.85) in hours, and days of hospitalization (4 ± 0.2 vs. 4.8 ± 0.3, *p* = 0.91) between the zinc and the placebo group, respectively, although a tendency was observed (Table 3). No deaths were reported in any of the participants of the study.

**Table 3:** Comparison of the time for improvement of clinical symptoms.

	**Zinc *n* = 50**	**Placebo *n* = 53**	***p***
Clinical improvement (hours), mean ± SE	76 ± 7	105 ± 8	0.01
Normalization of respiratory rate (hours), mean ± SE	37 ± 6	57 + 7	0.04
Normalization of O_2_ saturation (hours), mean ± SE	53 ± 7	87 ± 9	0.007
Normalization of respiratory distress (hours), mean ± SE	46 ± 4	56 ± 6	0.21
Normalization of temperature (hours), mean ± SE	6 ± 1	7 ± 2	0.85
Days of hospitalization, mean ± SE	4 ± 0.2	4.8 ± 0.3	0.91

*The children with pneumonia who received zinc supplementation improved in fewer hours in clinical status, respiratory rate, and oxygen saturation compared to the placebo group*.

No side effects were reported in children who received zinc or placebo.

### Immune Response

The lymphoproliferation to concanavalin A of the PBMCs from children with pneumonia measured by EdU Click-iT incorporation were comparable between the zinc (mean ± SE difference of intensity 89 ± 19) and the placebo group (136 ± 25, *p* = 0.14) before and after treatment (81 ± 20 vs. 93 ± 22, *p* = 0.7), respectively.

**Figure 3:**
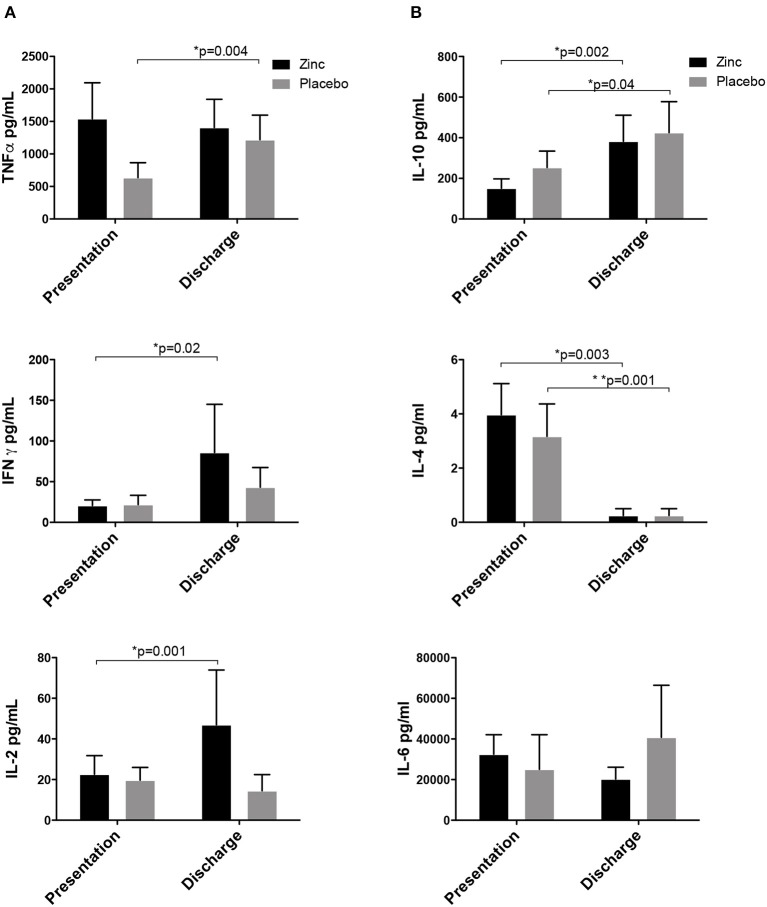
Cytokine responses of children with pneumonia supplemented with zinc or placebo. **(A)** TH1 cytokines: IL-2 and INF-γ increased in the zinc group, and TNFα increased in the placebo group, although in the zinc group, it remained high. **(B)** Th2 cytokines: IL-10 increased in both groups, IL-4 decreased, and IL-6 remained high in both groups.

Cytokine responses showed an increase in Th1 pattern for IL-2 (x ± SEM, 22 ± 9 to 46 ± 26) and INF-γ (x ± SEM, 20 ± 6 to 85 ± 59) after supplementation in the zinc group; TNFα (x ± SEM, 665 ± 267 to 1138 ± 359) increased in the placebo group, although in the zinc group, it remained high. From the Th2 cytokines, IL-10 increased in both groups (x ± SEM: zinc 151 ± 46 to 382 ± 128, placebo 253 ± 80 to 425 ± 152), IL-4 decreased (x ± SEM: zinc 3 ± 1 to 0.25 ± 0.2, placebo 3 ± 1 to 0.25 ± 0.25), and IL-6 remained high in both groups (Figure 3).

## Discussion

Zinc deficiency alters innate and adaptive immunity (8). Zinc supplementation improves immunity, ameliorates chronic dysfunctional inflammatory responses (7), and has been shown to shorten the duration and decrease severity in children with diarrhea, and since 2004, WHO and Unicef recommend zinc supplementation along with oral rehydration (10).

In the case of pneumonia, there are several trials and meta-analyses about zinc supplementation. Some found benefits (17–21, 24, 26, 31), others limited improvement (11), and some found a lack of benefits compared to placebo (12–14, 32). However, the outcomes and methodologies are different in each clinical trial, and there is no immunological and etiological assessment.

We conducted a randomized triple-blinded controlled clinical trial, using the recommended WHO dose of zinc for children with diarrhea in children with pneumonia; measuring the clinical symptoms as previously reported in many RCTs but with a translational approach where we measured cytokines, the lymphoproliferative capacity after zinc supplementation, and serum zinc levels; and detecting the etiological agents by multiplex PCR, trying to find a correlation of the pathogen, the clinical development, and the immune response.

We found that zinc improves oxygen saturation, tachypnea, and clinical status in fewer hours than the placebo group, independently of age, weight, height, BMI, nutritional, or vaccination status according to the covariance analysis, and this correlates with an increase in Th1 cytokines IFNγ and IL-2 in the zinc group at discharge of the patient (after zinc supplementation). Although there was no difference in hospital stay, the respiratory symptoms improved faster. Both hospitals attend to a very-low-income population, and the discharge of a patient depends on many factors; for example, some patients improved but stayed hospitalized to finish the antibiotic treatment due to the lack of resources to buy the antibiotic, or for administrative reasons like payment of the hospitalization and lack of money of the parents. We considered that hospital stay length is not a good clinical marker of improvement in our country, so we looked at other clinical markers, such as respiratory distress, oxygen saturation, or tachypnea, that denote the clinical improvement of the children. Viruses are responsible in up to 81% of the cases of pneumonia in children younger than 5 years old (33). Viruses are intracellular pathogens where a strong cellular immune response is needed, and in the case of both groups, we infer that an increase of proinflammatory cytokines TNFα and IL-6 and Th1 cytokines IFNγ and IL-2 took place between the time of entrance of the pathogen to the organism and the development of pneumonia, which is demonstrated by the lymphoproliferation detected at the moment of pneumonia and slight decrease at resolution when the blood draw was taken. A persistent level of TNFα and IL-6 at both times of detection was observed in both groups; it could be either the important systemic and pulmonary inflammation or the zinc deficiency associated with the production of proinflammatory cytokines such as IL-1β, IL-6, and TNFα (8, 34) because in both groups, serum zinc levels were below normal despite zinc supplementation at the moment of admission with pneumonia and at the discharge. It is noteworthy that TNFα showed a statistically significant increase in the placebo group, but the zinc group had high levels of TNFα at admission with pneumonia that persisted at discharge, comparable to the placebo group in the last measurement (Figure 2A). The increase in IFNγ and IL-2 with zinc supplementation compared to the placebo suggests a promotion of cell-mediated immunity over humoral immunity, also shown by a decrease in IL-4; this cell-mediated immunity could result in faster improvement of the clinical symptoms compared to the placebo group. IL-10, a down-regulatory cytokine, increased at resolution in both groups, which is expected after an inflammatory response takes place and where this cytokine in necessary to return to the basal state. In both groups, inflammation takes place due to the pathogens and the immune response, and it is demonstrated by a high level of proinflammatory cytokines and mononuclear proliferation in the basal blood sample. An explanatory model is shown in Supplementary Figure 1 based on the cytokine and lymphoproliferation responses according to the time of blood draws.

The children in the placebo group had a lower weight and height, which could be a confounding variable, although they were 5 months younger, and that would explain the differences; and it is noteworthy that malnutrition showed no differences between groups. Because of this difference in age, adjusting by age, weight, height, and BMI, the effect of zinc on the respiratory parameters with a covariance analysis did not show an effect, which means the younger age is not a bias in the better response of the zinc group. Another risk factor that was higher in the placebo group was incomplete vaccination schedule (50 vs. 30%, *p* = 0.04, placebo vs. zinc, respectively), although the results of the multiplex RT-PCR showed a very high proportion of patients with viral infections and co-infections that are not prevented by vaccines included in the Mexican vaccination program. The only virus detected by the PCR included in the National Vaccination program is influenza, and cases of influenza in both groups were comparable; also, *S. pneumoniae* is in the National Vaccination program, and both groups showed equal frequency. One limitation of the study was that the mothers did not provide the vaccination record; we could not analyze the differences in specific vaccines among groups and could not corroborate the vaccines received by each child.

Zinc levels increased in the treatment group compared to the placebo, but they were below normal levels at admission to the hospital for pneumonia and also at discharge despite the supplementation. It is possible that zinc was depleted by consumption by PBMCs or neutrophils because of the inflammatory response (34), or by the use of bacteria to drive key cellular processes during infection (35), but it is also possible that the children who attended both hospitals are from a very-low-income population, and zinc deficiencies due to malnutrition (in our group, up to 30%) could be the basal state of the children; a control of healthy subjects would have been suitable to answer these questions. Also, it is possible that a higher dose or a longer duration of treatment with zinc would have achieved a better serum zinc level, but we based it on the intervention described by WHO and other studies where the same dose of zinc showed benefits. It is noteworthy that the placebo group also presented a tendency to increase the zinc levels, although not significant; this could be related to the balanced food given at the hospital to very-low-income children.

Although we found many combinations of viruses and bacteria, the capacity to detect multiple bacteria in nasal washes is hard to interpret because only genetic material is detected by the multiplex PCR in the absence of a bronchial culture or blood culture, and the detection could be bacterial colonization, which has been described in up to 64% in the nasopharynx of healthy subjects (36). In the case of viruses, it is unknown if there is colonization or if they are only detected when inflammation and disease exist. No correlations of clinical, immunological, and etiological agents could be made due to the large number of combinations of viruses and/or bacteria. And the proportion of viral, bacterial, or combined detection was comparable between groups.

All the controversies about zinc supplementation for children with pneumonia are based on clinical trials where the zinc is administered and the clinical outcome is measured. We did not attempt to add clinical evidence in the context of a randomized controlled trial that has been extensively published but, rather, to look for immunological evidence of the effect of zinc in children with pneumonia. Some translational studies report immunological evidence of pathologies in humans in little as 5–10 patients; this study with 50 patients in each group adds immune evidence of zinc supplementation in children with pneumonia. This is the first study to provide immunological evidence of a Th1 response in the context of a randomized controlled clinical trial, where translational medicine is applied and where an immunological pattern is detected and correlates with the clinical outcome.

## Conclusions

Zinc supplementation showed benefits, shortening the duration of oxygen desaturation, tachypnea, and clinical symptoms in children with pneumonia, showing a Th1 response with the increase of IFNγ and IL-2 cytokines. Zinc supplementation along with the standard protocol for treatment could be further explored as a recommendation for children with pneumonia, which constitutes one of the most frequent causes of morbidity and mortality in children younger than 5 years old.

## Data Availability Statement

The datasets generated for this study are available on request to the corresponding author.

## Ethics Statement

The study was approved by the Institutional Review Boards of the Faculty of Medicine, Universidad Nacional Autónoma de México (036-2012), Hospital General de México (DI/13/505/05/026), and Hospital Pediátrico de Coyoacán (3020010114); written consent was obtained from parents or guardians. The procedures followed were in accordance with the ethical standards of the Helsinki Declaration.

## Author's Note

Previous affiliation of Rosa María Wong-Chew, Miguel Leonardo García-León, Jorge Luis Santiago-Cordova and Alejandra Pamela González-Rodríguez was Unidad de Investigación en Medicina Experimental, Facultad de Medicina, UNAM; Dr. Balmis # 148, Colonia Doctores 06726, México, D.F. The work was presented in part as abstracts at the annual meeting of the Asociación Mexicana de Infectología y Microbiología, San Luis Potosí, México, May 2015, Monterrey, México, May 2016, and the IX National Virology Meeting Puente de Ixtla, Morelos, México, November 2015.

## Author Contributions

RW-C: study design, data analysis, data interpretation, writing the manuscript, study coordination, writing a grant, and obtaining financial support for the study. JA-M, VF-R, and JS-C: patient recruitment, sample collection, and review of the manuscript. MG: study design, data interpretation, processing of the blood samples to determine the immune responses (lymphoproliferation and cytokine levels), and review of the manuscript. AG-R: processing of the nasal samples by the multiplex PCR methods to detect viral and bacterial pathogens and review of the manuscript.

### Conflict of Interest

RW-C reports personal fees from Sanofi Pasteur as a speaker and GSK and Abbvie for advisory boards, outside the submitted work. The remaining authors declare that the research was conducted in the absence of any commercial or financial relationships that could be construed as a potential conflict of interest.
