# Supplementary material for: Zinc Supplementation Promotes a Th1 Response and Improves Clinical Symptoms in Fewer Hours in Children With Pneumonia Younger Than 5 Years Old. A Randomized Controlled Clinical Trial
Source: Front Pediatr. 2019 Nov 14;7:431. doi: 10.3389/fped.2019.00431 (PMC6874056; doi:10.3389/fped.2019.00431)
Supplement: Supplementary file 2 [file Table_2.docx]

**Supplementary table 2.** **Comparison of viral agents single and co-detections by group.** There were no differences in the frequencies of viral or bacterial pathogens between groups.

| **VIRUS** | **ZINC** | | | **PLACEBO** | | |
| --- | --- | --- | --- | --- | --- | --- |
|  | **Single detection** | **Co-detection** | **Total** | **Single detection** | **Co-detection** | **Total** |
| **RSVA** | 3 | 8 | 11 | 3 | 3 | 6 |
| **RSVB** | 2 | 7 | 9 | 3 | 5 | 8 |
| **AdV** | 4 | 5 | 9 | 1 | 6 | 7 |
| **FluA** | 1 | 4 | 5 | 1 | 8 | 9 |
| **Flu B** | 0 | 3 | 3 | 0 | 2 | 2 |
| **HEV** | 1 | 6 | 7 | 1 | 5 | 6 |
| **HRV** | 3 | 5 | 8 | 6 | 4 | 10 |
| **MPV** | 2 | 13 | 15 | 1 | 10 | 11 |
| **PIV1** | 1 | 1 | 2 | 0 | 0 | 0 |
| **PIV 3** | 0 | 5 | 5 | 0 | 1 | 1 |
| **PIV4** | 0 | 1 | 1 | 0 | 2 | 2 |
| **HBoV** | 0 | 0 | 0 | 0 | 1 | 1 |
| **CoV OC43** | 0 | 3 | 3 | 0 | 1 | 1 |
| **Co V 229E** | 0 | 1 | 1 | 0 | 1 | 1 |
| **CoV NL63** | 1 | 0 | 1 | 1 | 1 | 2 |
| **Negative** | 11 | | 11 | 6 | | 6 |

Chi square 0.43.

RSVA, RSVB- Respiratory syncitial virus A or B, AdV-Adenovirus, Flu A or B- Influenza A or B, HEV- Human enterovirus, HRV- Human rhinovirus, MPV- Human metapneumovirus, PIV1, 3, 4- Parainfluenza virus 1, 3 or 4, HBoV-Human Bocavirus, CoV OC43, 229E or NL63- Coronavirus OC43, 229E or NL63.
