# Supplementary material for: Zinc Supplementation Promotes a Th1 Response and Improves Clinical Symptoms in Fewer Hours in Children With Pneumonia Younger Than 5 Years Old. A Randomized Controlled Clinical Trial
Source: Front Pediatr. 2019 Nov 14;7:431. doi: 10.3389/fped.2019.00431 (PMC6874056; doi:10.3389/fped.2019.00431)

Supplementary Figure 1. Correlation of cytokine detection and the development of disease in in children with pneumonia supplemented with zinc or placebo. At the moment of infection Th1 cytokines increase and promote lymphoproliferation (PBMC-peripheral blood mononuclear cells) and inflammation; during the acute phase of pneumonia IFNγ and IL-2 decrease, and at resolution an increase is observed with zinc supplementation in contrast to the placebo. TNFα remains high during pneumonia and at resolution in both groups, and IL-10 a downregulatory cytokine increases at resolution in both groups.

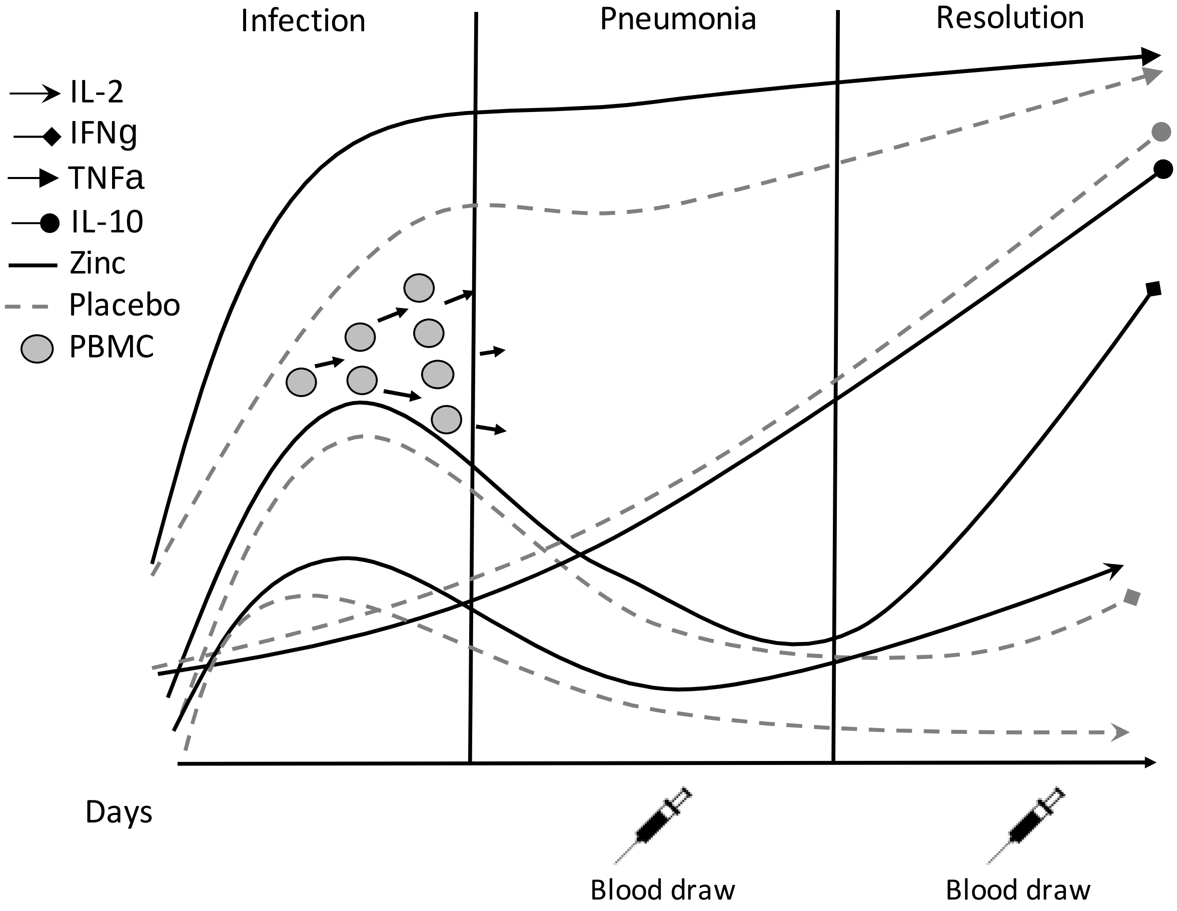

Supplement: Supplementary file 3 [file Image_1.pdf]
